# A behavioral economic risk aversion experiment in the context of the COVID-19 pandemic

**DOI:** 10.1371/journal.pone.0245261

**Published:** 2021-01-19

**Authors:** Bruno Kluwe-Schiavon, Thiago Wendt Viola, Lucas Poitevin Bandinelli, Sayra Catalina Coral Castro, Christian Haag Kristensen, Jaderson Costa da Costa, Rodrigo Grassi-Oliveira

**Affiliations:** 1 Developmental Cognitive Neuroscience Lab (DCNL), School of Medicine, Pontifical Catholic University of Rio Grande do Sul, Porto Alegre, Brazil; 2 Brain Institute of Rio Grande do Sul (BraIns), Pontifical Catholic University of Rio Grande do Sul, Porto Alegre, Brazil; Universidad Loyola Andalucia Cordoba, SPAIN

## Abstract

We investigated what degree of risk of infection with COVID-19 is necessary so that people intend to stay home, even when doing so means losing their salary. We conducted an online survey across Brazil during the initial outbreak, in which 8,345 participants answered a questionnaire designed to identify the maximum tolerated risk (k’) necessary for them to disregard social distancing recommendations and guarantee their salaries. Generalized linear mixed models, path analysis structural equation, and conditional interference classification tree were performed to further understand how sociodemographic factors impact k’ and to establish a predictive model for the risk behavior of leaving home during the pandemic. We found that, on average, people tolerate 38% risk of infection to leave home and earn a full salary, but this number decreased to 13% when the individual risk perception of becoming ill from severe acute respiratory syndrome coronavirus-2 is considered. Furthermore, participants who have a medium-to-high household income and who are older than 35 years are more likely to be part of the risk-taking group who leave home regardless of the potential COVID-19 infection level; while participants over 45 years old and with good financial health are more likely to be part of the risk-averse group, who stay home at the expense of any salary offered. Our findings add to the political and public debate concerning lockdown strategies by showing that, contrary to supposition, people with low socioeconomic status are not more likely to ignore social distancing recommendations due to personal economic matters.

## Introduction

Risk perception refers to the subjective assessment that people make regarding the characteristics and severity of a perceived threatening stimulus or situation, including knowledge of the risk of contagion and disease severity [1]. Risk behaviors include actions that underlie a goal with uncertainty regarding its outcomes, such as negative costs or possible benefits [2]. Both risk perception and risk behaviors are closely associated with the motivation to embrace self-preservative care in situations such as disease outbreaks [3].

Throughout the COVID-19 outbreak, a key strategic plan adopted by many governments was strict quarantine and lockdown of the general population to reduce and prevent community transmission of the new virus [4, 5]. The social distancing recommendations aimed to minimize overloading of hospitals by demanding that people remain at home and alone as much as possible (except to procure food/basic supplies and medical needs). Recent data support the effectiveness of social distancing in slowing the progression and spread of the new coronavirus [6, 7]. However, although it has been shown that implementing social-distancing measures early on proved to be effective, reducing the fatalities due to the deceleration of the contagion rate [8], an important political and public debate has arisen since the beginning of the lockdowns.

Briefly, it has been suggested that this approach will be unsustainable in the long-term, particularly because shutting down the whole population of a given country could generate adverse consequences for the economy [9]. Therefore, although social distancing is containing the ever-growing number of COVID-19 cases in Brazil [10–12], the current Brazilian president has demanded an end to lockdown strategies, to alleviate the ongoing global economic crisis [13]. One of the key points in his decision regards the assumption that many citizens are not capable of adapting to home-office strategies, and many may lose their jobs, or face severe income drawbacks. Consequently, people with low socioeconomic status may face more threat during the quarantine period, including scarcity of basic resources, such as food [14, 15]. Moreover, the inconsistency in implementing social distancing recommendations in Brazil has also been based on a second widespread assumption [14], which is that the threat and risk of severe negative health outcomes due to COVID-19 are minimal, potentially affecting people who are at higher risk for severe illness (e.g., older people and people with pre-existing medical conditions). Nevertheless, at the individual level, this dilemma, which could be rephrased as staying home with no risk of infection but possibly not having sufficient resources, or maintaining an out-of-work routine that allows them to maintain their resources but increases the possibility of infection, is particularly complex given that socioeconomic factors could interact with the perception of health benefits attributed to social isolation measures [10].

Therefore, the aim of this study is three-fold: (a) to investigate what degree of risk of infection with COVID-19 is necessary, on average, so that people intend to stay home, even when doing so means losing their salary; (b) to investigate which sociodemographic factors might influence the maximum tolerated contamination risk of COVID-19 to keep an adequate and an unfavorable salary offer; and (c) to establish a predictive model for risk-taking and risk aversive behaviors during the COVID-19 pandemic. To achieve these goals, we generated an online survey in which participants had to answer an experimental COVID-Risk Aversion Questionnaire. Hence, considering that policy makers need to focus on bringing awareness and social restraint among people rather than going for stringent lockdown measures, exclusively [16]; and that social distancing of the entire population, case isolation, household quarantine and school and university closure is predicted to have the largest impact, short of a complete lockdown which additionally prevents people going to work [17]; the ultimate goal of this study is to contribute to public policies in the context of the COVID-19 pandemic, for example, by identifying possible target groups for educational campaigns that can sensitize people regarding the importance of social distancing and overall self-care.

## Methods

### Ethical information

The data used in the present study were collected between April 18–May 11, 2020 via an online survey using Qualtrics, a full-featured, web-based tool for creating, distributing, and capturing online surveys. Any Brazilian over 15 years of age, residing in Brazil or abroad could complete the survey by accessing a link made available on different social networks on the internet. Survey participants were invited to complete the online questionnaire anonymously and voluntarily; in the case of minors (18 years old), legal guardians were asked to sign the Free and Informed Consent Form, showing awareness of their children’s participation in the research. Participants were not compensated for their participation. This survey was approved by the National Committee for Ethics in Research (CONEP, 30502620.4.0000.0008).

### Sampling plan

All participants were recruited through electronic media (e.g., social media, websites, blogs, etc.) and a snowball sampling method, whereby the researcher invited the participants to share the survey with their contacts. The sample size was calculated using the public domain program OpenEpi (www.openepi.com), adopting a 95% confidence level, a 1% margin of error, and a random sample. Considering that approximately 127 million people in Brazil have internet access, we estimated an initial sample size of 5,670 participants.

Furthermore, a series of criteria was applied to maximize data reliability. Initially, we excluded participants who took less than five minutes to complete the survey. Then, regarding the socioeconomic variables, we excluded participants who provided invalid information on age, ZIP-Code, and the final four numbers of their cell phones (only the final four numbers were asked to avoid identifying the participant). Afterwards, because we were not interested in investigating changes within participants over time, possible repeated measures were excluded by checking both repeated ZIP-Codes and the final four numbers of participants’ cell phones. Concerning the experiments, only data from participants who completed the full study and who performed the experiments with 100% reliability were analyzed. To check the reliability of the experiment data, we followed an axiomatic neuroeconomic approach [18] that excluded participants who did not follow their own preferences. For instance, a participant who chose to leave home with a 5%, 10%, or 15% risk of infection and to stay home with a 25%, 35%, 50%, and 60% risk of infection, could not choose to leave home when an 80% risk of infection was presented. Moreover, since this was an online study in which participants could enroll at any given moment, experimenters were blind to participants’ moderators and outcomes. Likewise, the researchers who processed and analyzed the data were blind to participants’ choices and moderators.

### Experiment design

Our experimental COVID-19 Risk Aversion Questionnaire was designed at two levels. At the first level, all participants were requested to answer questions regarding their average monthly household income, their financial health, and their risk perception.

Precisely, participants were initially requested to give the range of their family income so that the monetary reward offered to each participant represented approximately 100% (adequate full salary offer) or 50% (unfavorable half salary offer) of the average salary of the selected range. This strategy was used to minimize the effects of a marginal utility function, avoiding offering a financial reward much higher or lower than the average financial *status quo* of the individual. Based on Brazilian socioeconomic reality, participants were able to choose, from eight different ranges, the range that best represented their average family income (S1 File). Next, considering that risk perception might differ for each participant, a 0–100 scale was included, and participants were requested to indicate the perceived risk of becoming severely ill if infected with COVID-19. Then, to investigate the state of a participant’s financial health (i.e., a construct that includes personal perception of need for money and monetary affairs) [19] we added two 0–100 scales to investigate the proportional amount of savings each participant had at the end of a regular month (i.e., money savings) and how much each participant feared that their savings would not last (i.e., money duration). Both items were taken from the Consumer Financial Protection Bureau (CFPB) Financial Well-Being Scale that was designed for practitioners and researchers to accurately and consistently quantify the extent to which someone’s financial situation provide them with security and freedom of choice [19–21], thus, working as a proxy for personal perception for money need. Then, to estimate the financial health, an index was obtained as follows: (100-“Savings” + “Duration”)/2. Thus, in our index 0 depicts 0% of financial health (the worse financial health, in which participants struggled to save money and/or believed that they do not save enough) and 100 depicts 100% of financial health (the best financial health, in which participants were able to save money and/or were not worried that their savings would not last).

Once the first level had been achieved, all participants were requested to answer 16 items about their preference for two alternatives differing in both monetary reward and risk of infection. Hence, at the second level, participants had to choose between leaving home for an amount of salary while being exposed to a given amount of infection risk or to stay home with no salary and no infection risk. For instance, a person with a family monthly income of between R$3000.00 to R$5000.00 (approximately $1,367.48 to $2,279.14, according to the purchasing power parity function and based on The World Bank) would be asked the following question: What would you choose? (a) to leave home for a salary of R$3999.00 while being exposed to a 5% risk of infection with COVID-19 or (b) to stay home with no salary and no risk of infection with COVID-19. Importantly, the 16 items on COVID-19 risk aversion were hypothetical, that is, participants made the decision without the perspective of obtaining real money. However, as shown by Brañas-Garza et al. (2020) hypothetical rewards are a good alternative to real rewards and paying or not for the measurement of risk preferences produces the same findings [22].

In this questionnaire, the precise values associated to 100% (varying from 99.9% to 99.4%) and 50% (varying from 49.9% to 49.4%) of the average salary of the selected range were never offered to the participants to guarantee the variability in the collected data. Because the chance of infection could vary between eight possibilities (5%, 10%, 15%, 25%, 35%, 50%, 65%, and 80%) and the amount of monthly income offered could vary between two possibilities (approximately 50% and approximately 100% of the average salary of the selected range), 16 items were randomly assigned for each participant. This questionnaire has a mixed design with a within-individual factor accounting for the probability of infection by COVID-19 and a between-individual factor accounting for the monthly income offered.

To test whether the proportion of the frequency of each question was equally balanced between the household income ranges among all the 16 items a multivariate analysis of variance (MANOVA) was performed, afterwards. The dependent variables were the proportions of the frequencies in which each item appears among the 16 "positions", and the independent variables were the eight chances of infection, the two salary offers, the six household income ranges, and their interactions. Since the MANOVA revealed no significant main effect for the chance of infection (*p* = .453), salary (*p* = .191), household income (*p* = .129) neither their interactions (chance of infection and salary, *p* = .615; chance of infection and household income, *p* = .072; salary and risk, *p* = .169; chance of infection, salary, household income, *p* = .361), we could infer that the means and the variances of the frequencies in which each item appears among the 16 "positions" were balanced across the independent variables. Furthermore, as a validation analysis to certify that people behaved as expected on the experimental COVID-Risk Aversion Questionnaire, i.e., decreasing the preference to leave home when the chances of contamination increase and preferring to leave home to obtain the full salary offer in comparison to the half salary offer, a series of generalized linear mixed models were performed and shown in the (**S2 Table in S1 File**).

### The k’ estimate

Much like other discounting paradigms [23], which are designed to identify the indifference point—and which reflect equal preference for two dichotomous reward alternatives differing in both delay and magnitude—in our study, the indifference point “*k*” (*k’* estimate) depicts the maximum risk of COVID-19 infection that participants are willing to tolerate in order to leave home and guarantee their salaries. To extract the *k’* estimate, we initially ordered, for each subject and salary offered, the eight COVID-19 risk aversion items according to the risk of being infected, from the less risky scenario (5% risk of infection) to the riskier scenario (80% risk of infection). The non-risk choice of staying home was coded 0 and the risk choice of leaving home was coded 1. We assumed that the *k* for each participant would be the final risk choice he/she made. For instance, a participant who chose to stay home when the risk of infection reached 35%, might have also chosen to stay home when the risk of infection was 5%, 10%, 15%, or 25%; but might not have chosen to stay home when the risk of infection was 50%, 65%, and 80%; therefore, we coded these options as 1, 1, 1, 1, 0, 0, 0, 0. Participants who only chose non-risk options were presumed to have a *k* of 0%, since they would not tolerate any risk of infection. Participants who only chose risk options were presumed to have a k of 80%, since this was the maximum risk of infection that we asked them to hypothetically face and we had no additional information to estimate that the *k* might be higher. This neuroeconomic axiomatic approach, which maintained a close connection between theoretical constructs and empirically observable phenomena [18], was only possible when 100% rational answers were included, in which participant preferences were consistently fulfilled and, therefore, reliable.

### Analysis plan

To achieve our three main goals, our data analysis was composed of four different steps according to the study aims, as shown in **S1 Fig in S1 File**.

In the first step, to achieve the first goal and investigate what degree of risk of infection with COVID-19 is necessary, on average, so that people intend to stay home, even when doing so means losing their salary we first perform a series of descriptive statistic to obtain frequencies, central tendencies and dispersions. The difference between *k’* estimates at half and full salary offers, before and after adjusting for the risk perception were tested with Student t-test and effect sizes were calculated by Pearson's correlation coefficient (r < 0.10 small effect size; r < 0.30 medium effect size; r < 0.50 large effect size) [24].

Afterwards, to achieve our second goal and to investigate which sociodemographic factors might influence the *k*’ estimate a path analysis structural equation modeling (SEM) was performed. The SEM is a useful analysis to describe both the direct and the indirect effects among a set of variables taking into account all significant correlations between predictive variables and *k’* estimates in a covariance matrix [25]. In other words, SEM takes into account that some sociodemographic variables play a dual role of simultaneously being dependent on one or more variables, while acting as independent variables in that they influence others [26, 27]. Age, sex, years of education, COVID-19 risk group classification, potential COVID-19 contact, current flu symptoms, being a health-related professional, and *k* estimates were included in the SEM. Potential COVID-19 contact was obtained by adding together the four dichotomous variables: i) participant’s belief about being infected by COVID-19, ii) past or current confirmed diagnosis of COVID-19, iii) participant’s belief about being close to someone infected by COVID-10, and iv) a relative’s past or current confirmed diagnosis of COVID-19. Respiratory disease related variables were recent flu symptoms (yes or no) and number of criteria each participant fulfils for COVID-19 risk group, i.e., the sum of criteria that defines the risk groups (being older than 60 years, respiratory disease, cardiovascular disease, obesity, diabetes, liver disease, cancer, HIV/AIDS, other immune related diseases, rheumatoid arthritis).

Then, to achieve our third goal, we initially explored how the sociodemographic variables may contribute to the probability of being classified as extreme risk-taking (those participants who always chose to leave home even for a half salary offer, “RT”) and extreme risk averse (those participants who always chose to stay home even at the expense of a full salary offer, “RA”). A backward and forward stepwise logistic regression was executed for each one including all sociodemographic variables that have been taken into account in the prior SEM analysis, was implemented in this regard. Afterwards, a conditional inference tree model was generated using a variable-selection procedure through the significant predictors evidenced in our SEM, exploring potential network types among these variables and generating groups that are maximally different from each other. By using a conditional inference tree, we were able to establish two predictive models and identify the participants’ profiles most likely to always choose to leave home even for a half salary offer (i.e., being classified as RT group) and the participants’ profiles most likely to always stay home even at the expense of a full salary offer (i.e., being classified as RT group).

All analyses were performed using the open-source statistical software R (version 4.0.0). The “sem” function from the “lavaan” package was used to generate a path analysis structural equation. The conditional inference models were achieved using the “ctree” function from the package “partykit,” which provides tree-structured regression models that deal with different types of variables and that do not rely on distributional requirements [28, 29]. A quadratic-type test statistic was applied for variable selection and the stopping criterion was based on Bonferroni adjusted p-values. The “confusionMatrix” function from the “caret” package was used to obtain an overview of the accuracy statistics [30].

## Results

### Sample characteristics

A total sample of 8,345 participants completed the experimental COVID-19 Risk Aversion Questionnaire, but 13.5% of these were not included in the analysis because they did not fulfill the 100% axiomatic reliability criteria necessary to obtain the *k’* estimate (see methods section). Therefore, a total of 7,216 participants were considered for the analysis. As **Table 1** reveals, most of our sample comprised individuals between 26–45 years old, predominantly women and with a high educational level. Curiously, approximately one-third of the sample worked as health professionals or their main area of expertise was directly related to health (e.g., university professors of health-related disciplines).

**Table 1:** Sample characteristics.

	Total sample (n = 7,216)
*Demographics*	
Age, years, mean (SD)	38.45 (12.7)
Age, years range, n (%)	
15–25	1211 (16.7)
26–35	2153 (29.8)
36–45	1874 (25.9)
46–55	1060 (14.6)
≥56	918 (12.7)
Sex, n (%)	
Female	5412 (75)
Male	1804 (25)
Unemployment status, n (%)	266 (3.6)
Health-related professional, n (%)	2170 (30)
Education, n (%)	
Secondary	778 (10.7)
Undergraduate	2360 (32.7)
Graduate	4078 (56.5)
Flu-like symptoms, n (%)	632 (8.7)
Yes/no risk group for SARS-CoV-2, n (%) ^a^	2271 (.31)
Yes/no potential COVID-19 Contact, n (%) ^b^	2083 (28.8)
*COVID-Risk Aversion Questionnaire*	
Household income, n (%) ^c^	
<R$3,000 (Lower low class)	1155 (16)
R$3,001—R$5,000 (Upper low class)	1206 (16.7)
R$5,001—R$7,000 (Lower middle class)	1029 (14.2)
R$7,001—R$10,000 (Upper middle class)	1124 (15.5)
R$10,001—R$15,000 (Lower high class)	1070 (14.8)
>R$15,001 (Upper high class)	1632 (22.6)
Money savings, mean (SD) ^d^	44 (38)
Savings not last, mean (SD) ^e^	45 (35)
Financial health index, mean (SD) ^f^	50 (31)

Note.

a) Number of participants that fulfils, at least, one criterion for risk group for SARS-CoV-2.

b) To present a more informative description of the sample, here we described the dichotomized version of the variable “Potential COVID-19 contact”. All participants who believe about being infected by COVID-19 or had/have past/current confirmed diagnosis of COVID-19 or believe about being close to someone infected by COVID-10 or had/have a relative’s past/current confirmed diagnosis of COVID-19 were classified as “yes, had potential COVID-19 contact”. Participants who reported no for all these questions were coded as “no, did not have potential COVID-19 contact”.

c) For a matter of comparison with USA dollars using purchasing power parity function and based on The World Bank, household income can be estimated as following: R$3,000 = $1,367.48, R$5,000 = $2,279.14, R$7,000 = $3,190.79, R$10,000 = $4,558.28, R$15,000 = $6,837.41. As reported in the method section

d) refers to the proportional amount of savings each participant has in the end of a regular month (savings)

e) refers to the how much each participant fears that their savings would not last (duration); and

f) refers to the financial health index, which was calculated as (100-“Savings not last” + “Money savings”)/2. The financial health index describes the state of one's personal monetary affairs, hence, 0 depicts 0% of financial health and 100 depicts 100% of financial health. V = Cramer's V effect size; r = Pearson r effect size.

**Fig 1:**
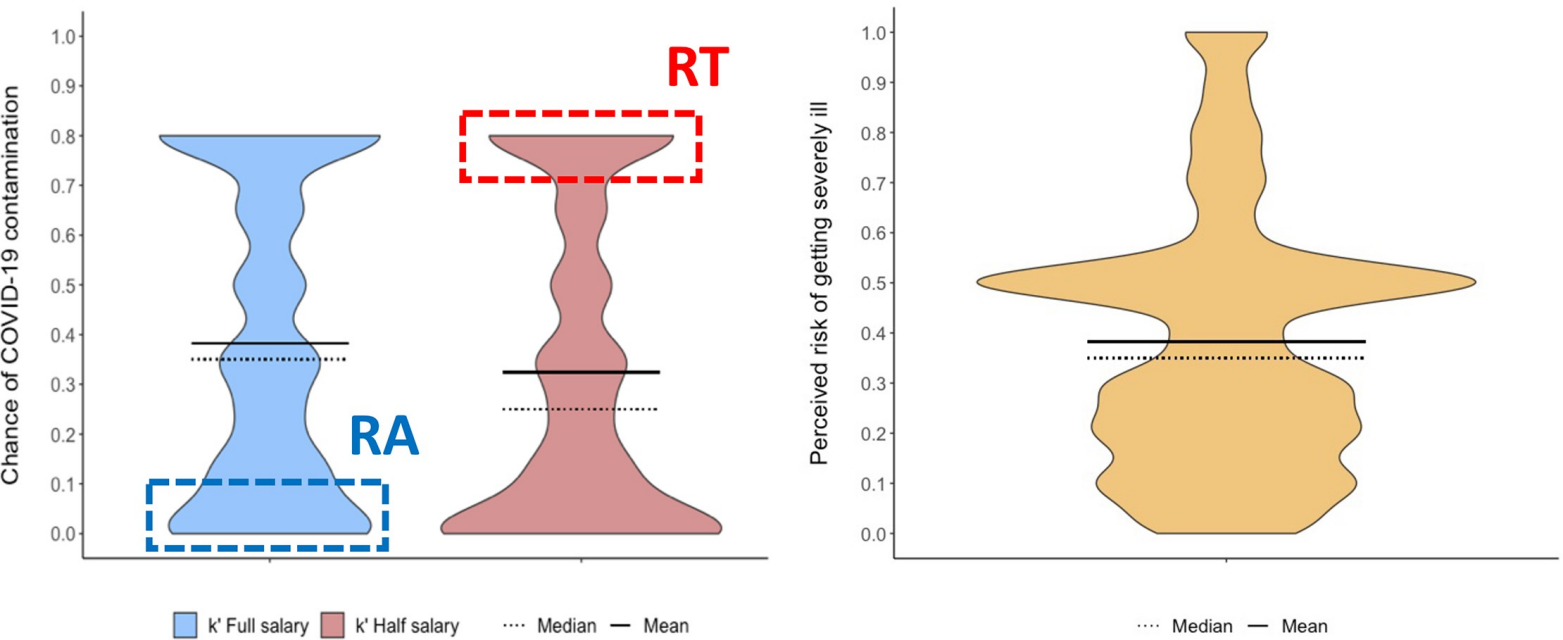
Violin plots in which the horizontal axis depict the density of data distribution. (A) k’ estimate; RA, extreme risk-averse participants (participants always who chose to stay home even at the expense of a full salary offer); RT, extreme risk-taking participants; (participants who always chose leave home even for a half salary offer); (B) Perceived risk of getting severely ill.

To elucidate stay or leave home decision-making processes during the COVID-19 pandemic, we first examined the average *k’* estimates for the entire sample (**Fig 1**). We observed that people tend to disregard social distancing recommendations whenever the chance of COVID-19 infection is lower than 32% (SD = .32, median = .25) and 38% (SD = .31, median = .35) to obtain half and full salary, respectively. This difference proved to differ significantly (t = -8.08, *p* < .0001, r = .09). Interestingly, regardless of the salary offered and the manipulated chance of COVID-19 infection, 18% (n = 1341) of our sample would always choose to stay home at the expense of their salaries (see bottom portion of **Fig 1A**), while 21% (n = 1550) would always choose to leave home to guarantee their monthly income (see top portion of **Fig 1A**). We also observed that the top and bottom proportions of **Fig 1A** changed according to the salary offered, suggesting that, for a half-salary offer, the number of participants that would opt to stay home without any salary increases. In other words, these participants would not expose themselves to the chance of COVID-19 infection if they would receive less than their regular salary.

Next, we examined individual risk perception of becoming severely ill if infected by COVID-19. **Fig 1B** shows that, in general, the mean risk perception was 38% (SD = .26, median = .35). Nevertheless, 58% (n = 4189) of the sample perceived the risk of becoming severely ill as lower than 50%, while 23% (n = 1669) perceived the risk as higher than 50%. Furthermore, as clearly observed in **Fig 1B**, a considerable number of participants (approximately 19%, n = 1358) perceived the risk of becoming severely ill as exactly 50%; however, one could hypothesize that some of those participants were unable to estimate their own risk of becoming severely ill and, thus, reported it as half-half. Then, when the *k’* estimate was adjusted based on the risk perception by multiplying one by the other (i.e., the probability of both events, being contaminated and becoming severely ill, occur), our data revealed that participants are willing to tolerate a 11% and 13% chance of becoming severely ill if contaminated by COVID-19 to obtain half and full salary, respectively. Such adjustment in the tolerated risk based on the risk perception proved to be significantly different from the non-adjusted tolerated risk of contamination for both half (t = 35.69, *p* < .0001, r = .44) and full (t = 43.60, *p* < .0001, r = .51) salary offers. Finally, a cumulative proportion of staying home answers was calculated based on the *k*’ estimate considering the by the sum of staying home answers divided by the total amount of answers (**S2 Fig in S1 File**). For instance, a participant who chose to stay home when the risk of infection reached 35%, might have also chosen to stay home when the risk of infection was 5%, 10%, 15%, or 25%; but might not have chosen to stay home when the risk of infection was 50%, 65%, and 80%; so, his/her staying home answers were accounted for 5%, 10%, 15%, 25% and 35% risk of COVID-19 contamination.

### The role of sociodemographic factors in k’ estimates

Nevertheless, one could argue that both the risk perception and *k’* estimates probably depend on several sociodemographic factors, such as financial health, educational level, being a health professional, being part of the risk group for COVID-19, etc. For instance, it is acceptable to hypothesize that someone who does not have good financial health (i.e., who does not save enough money by the month end and fears that savings will not last) might have a higher *k’* estimate, since such a participant needs to leave home to guarantee any salary offer. Considering the decision to leave home as a risk behavior, this idea is supported by evidence that the rate and burden of risk behaviors are affected by socioeconomic status, particularly for people living in low- and middle-income countries [31, 32]. On the other hand, an epidemiologist may have a more accurate risk perception regarding the odds of becoming severely ill if infected by COVID-19 than a restaurant owner or taxi driver. Therefore, a SEM was established to gain further insight into the role of sociodemographic factors and to provide a possible structural relationship to explain how such variables may directly or indirectly influence the *k*’ estimate. The predictors included in the SEM were: financial health, educational level, sex, age, household income, potential contact with someone with COVID-19, being a health professional, and being part of the risk group for COVID-19.

**Fig 2:**
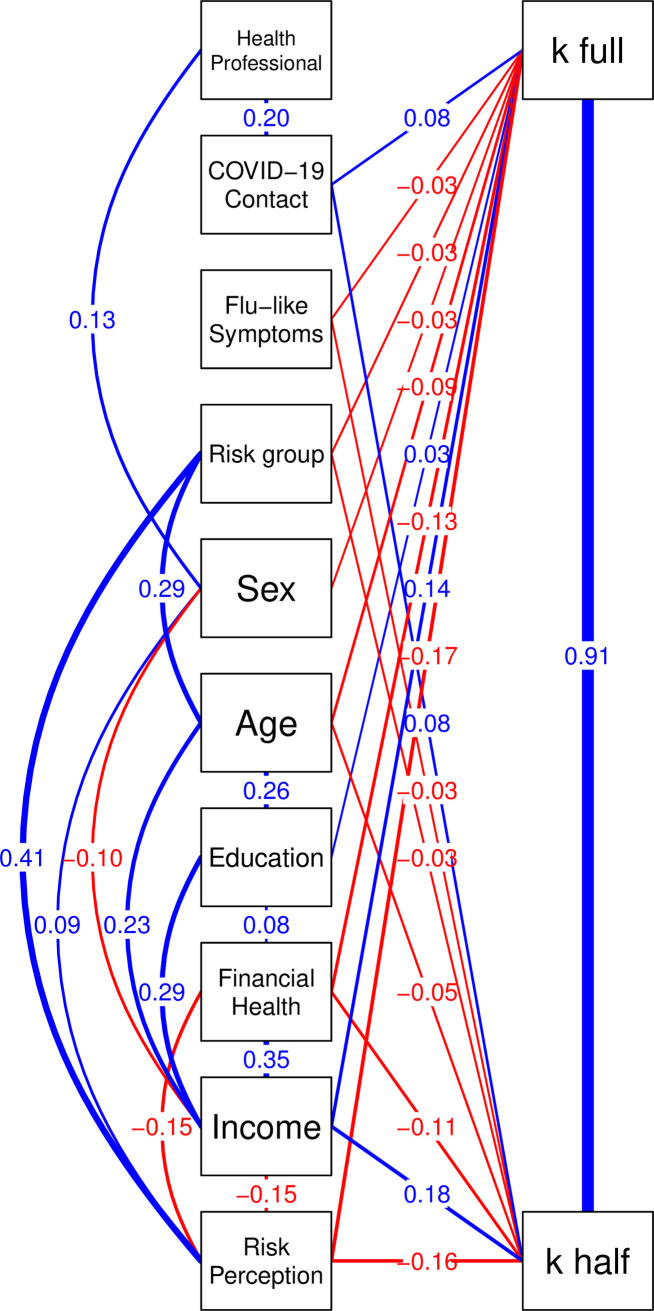
Structural Equation Model (SEM). All the significant parameter estimates were standardized so they can be interpreted with reference to other parameters in the model and relative strength of pathways within the model.

The SEM results revealed that our model was supported with the following fit indexes within the expected range [χ^2^(32)  = 655.44, *p* < .000; CFI  =  .97; TLI  =  .93; RMSEA  =  .052, confidence interval [CI] 95%  =  .049, .055] (**Fig 2**). Indeed, the SEM confirmed that for both k’ estimates, household income was a positive predictor while risk perception was a negative predictor. Moreover, the financial health index, age, and being in a COVID-19 risk group were negative significant predictors of both *k’* estimates, while COVID-19 potential contact was significantly associated with both *k’* estimates, suggesting that people who report higher *k’* estimate (and, therefore, tolerate higher risks of infection) have a higher chance of being in contact with someone who may be infected. Although this last result may sound contra intuitive, at first, it might turn that people who tolerate more risk are those people that must leave home more often for any reason, consequently increasing the odds of being in contact with someone who may be infected, explaining the positive relationship. Overall, these results imply that although individual risk perception and income are important factors that influence the risk participants are willing to take for their full or half salaries, additional financial and sociodemographic factors are also important in this relationship. Finally, we observed that sex and education have a specific effect on *k’* estimate when participants received the full salary offer. More precisely, we found that being a woman tend to negatively impact the tolerated risk to obtain the full salary offer, only. On contrary, education exerts a positive effect on the full salary offer. Curiously, both effects are not significant for half salary offer.

### Predictive models for extreme risk-taking and extreme risk averse behavior

In the context of COVID-19, our evidence suggests that the decision to stay home at the expense of a salary is a multifactorial phenomenon that depends, mainly, on risk perception and financial factors. Nevertheless, a predictive understanding of how such an individual dilemma could be conceived within the context of possible public health policies has not yet been achieved. Hence, bearing in mind that approximately 40% of our sample had already made their decision a priori, regardless of the manipulated chance of COVID-19 infection, we identified two groups that require special attention: extreme risk-taking participants (“RT”) who would always choose to leave home even for a half salary offer (top portion of the red violin plot, **Fig 1A**), and extreme risk-averse participants (“RA”) (bottom portion of the blue violin plot, **Fig 1A**).

**Fig 3:**
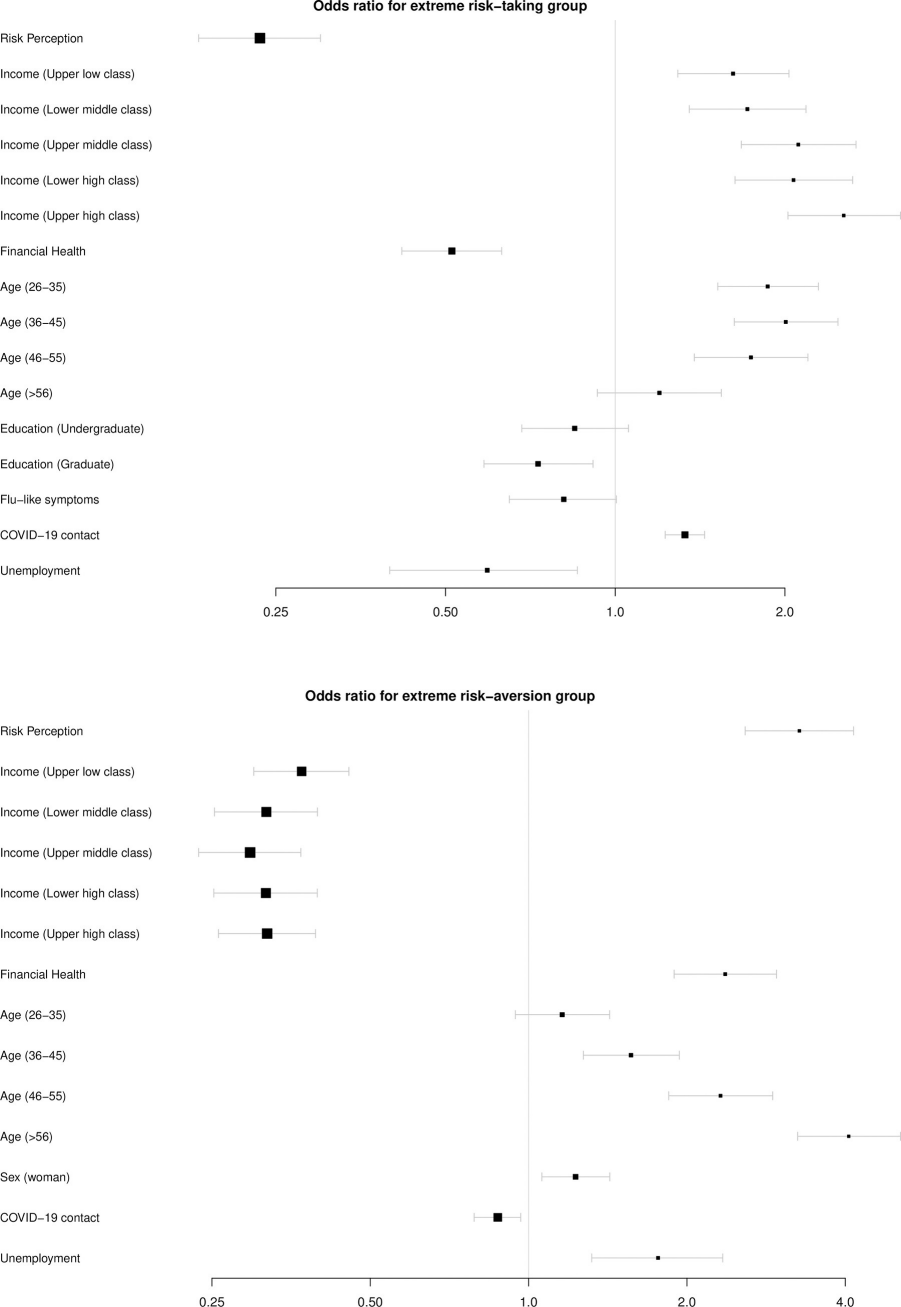
Odds ratio of the generalized linear model. Confidence intervals are set for 2.5% and 97.5%.

The results were noteworthy (**Fig 3**). First, the logistic regression revealed that for the RT group we found that the effect of the income systematically grows from the lower class to the higher class, suggesting that as more money a participant has more likely to be part of the RT group (**Fig 3A**). This data corroborates the effect of financial health, which suggests that as greater the financial health is less likely a participant will be classified as RT group. Another interesting finding relies on the effect of age. The higher effect is observed in the 36 to 45 years old range and the overall effects decrease as the age range increases, and no significant effect was observed for the elderly group. Concerning education, a significant effect was found for the graduate group, only, suggesting that higher education increases the odds of being classified as RT. As already shown in the SEM analysis, an important effect was found for risk perception, and a significant effect for COVID-19 contact was also found. Although the stepwise method suggested that the best model includes flu-like symptoms, no significant effect was found for this predictor.

Concerning the RA group (**Fig 3B**), our findings were in the same direction. First, we found an important effect for risk perception, suggesting that as much risk someone perceived of getting severely ill more likely that this participant will be classified as RA. The effects of income go in the opposite direction and, overall, income has a negative effect in the odds of being classified as RA. Remarkably, the effects of income were “C” shaped with the stronger ones observed for both the upper lower classes and for both the higher classes, and the weaker effect observed in the middle-class group. Additionally, the effect of age was stronger for the older groups, suggesting that as higher the age higher the chances of being classified as RA. An interesting effect for sex was found, suggesting the being a woman increases the chance of being classified as RA. As expected, a negative effect was found for COVID-29 contact.

Finally, a training and testing sample were randomly assigned at 70% and 30% of the total sample, and two independent predictive models were built (for a comparison between the RT and RA groups in the training sample, see **Table 2**). The first conditional inference model aimed to predict the RT group and, therefore, all the significant predictors found in the SEM for the full salary offer were included, in addition to the *k’* estimate at the half salary offer. The second conditional inference model aimed to predict the RA group and all the significant predictors found in the SEM for the half salary offer were included in addition to the *k’* estimate at the full salary offer.

**Table 2:** Demographics characteristics of the sample.

	Samples		Test (df)	Effect size	p-value
	RA (n = 977)	RT (n = 1,134)			
*Demographics*					
Age, years range, n (%)			χ^2^ = 88.46 (4)	*V* = .09	**< .0001**
15–25	121 (12.3)	128 (11.2)	χ^2^ = 0.60 (1)	*V* = .01	.435
26–35	235 (24)	365 (32.1)	χ^2^ = 17.06 (1)	*V* = .05	**< .0001**
36–45	220 (22.5)	348 (30.6)	χ^2^ = 17.81 (1)	*V* = .05	**< .0001**
46–55	166 (16.9)	179 (15.7)	χ^2^ = 0.55 (1)	*V* = .01	.455
≥56	235 (24)	114 (10)	χ^2^ = 74.5 (1)	*V* = .12	**< .0001**
Sex, n (%)			χ^2^ = 6.06 (1)	*V* = .03	**.013**
Female	766 (78.4)	837 (73.8)	-	-	-
Male	211 (21.5)	297 (26.1)	-	-	-
Unemployment status, n (%)	60 (6.1)	17 (1.4)	χ^2^ = 32.18 (1)	*V* = .07	**< .0001**
Health-related professional, n (%)	243 (24.3)	382 (33.6)	χ^2^ = 19.56 (1)	*V* = .06	**< .0001**
Education, n (%)			χ^2^ = 8.66 (2)	*V* = .03	**.013**
Secondary	131 (13.4)	100 (8.8)	χ^2^ = 6.22 (1)	*V* = .04	**.012**
Undergraduate	300 (30.7)	344 (30.3)	χ^2^ = 0.87 (1)	*V* = .00	.349
Graduate	546 (55.8)	690 (60.8)	χ^2^ = 5.90 (1)	*V* = .03	**.015**
Flu-like symptoms, n (%)	83 (8.4)	90 (7.9)	χ^2^ = 2.17 (1)	*V* = .00	.640
Yes/no risk group for SARS-CoV-2, n (%) ^a^	429 (43.9)	295 (26)	χ^2^ = 74.591 (1)	*V* = .12	**< .0001**
Yes/no potential COVID-19 Contact, n (%) ^b^	235 (24)	410 (36.1)	χ^2^ = 36.22 (1)	*V* = .08	**< .0001**
*COVID-Risk Aversion Questionnaire*					
Household income, n (%) ^c^			χ^2^ = 155.84 (5)	*V* = .10	**< .0001**
< R$3,000 (Lower low class)	264 (27)	107 (9.4)	χ^2^ = 153.33 (1)	*V* = .14	**< .0001**
R$3,001—R$5,000 (Upper low class)	149 (15.2)	189 (15.8)	χ^2^ = 1.54 (1)	*V* = .00	.214
R$5,001—R$7,000 (Lower middle class)	112 (11.4)	151 (13.3)	χ^2^ = 3.01 (1)	*V* = .01	.082
R$7,001—R$10,000 (Upper middle class)	120 (12.2)	190 (16.7)	χ^2^ = 9.83 (1)	*V* = .04	**.001**
R$10,001—R$15,000 (Lower high class)	131 (13.4)	186 (16.4)	χ^2^ = 4.16 (1)	*V* = .02	**.041**
> R$15,001 (Upper high class)	201 (20.5)	320 (28)	χ^2^ = 17.97 (1)	*V* = .05	**< .0001**
Money savings, mean (SD) ^d^	43 (38)	43 (38)	W = 559191	r = .00	.706
Savings not last, mean (SD) ^e^	42 (35)	45 (37)	W = 529910	r = .02	.083
Financial health index, mean (SD) ^f^	51 (31)	49 (31)	W = 570504	r = .01	.235
Perceived risk, mean (SD)	.46 (.29)	.32 (.25)	W = 718174	r = .16	**< .0001**

Note.

a) Number of participants that fulfils, at least, one criterion for risk group for SARS-CoV-2.

b) To present a more informative description of the sample, here we described the dichotomized version of the variable “Potential COVID-19 contact”. All participants who believe about being infected by COVID-19 or had/have past/current confirmed diagnosis of COVID-19 or believe about being close to someone infected by COVID-10 or had/have a relative’s past/current confirmed diagnosis of COVID-19 were classified as “yes, had potential COVID-19 contact”. Participants who reported no for all these questions were coded as “no, did not have potential COVID-19 contact”.

c) For a matter of comparison with USA dollars using purchasing power parity function and based on The World Bank, household income can be estimated as following: R$3,000 = $1,367.48, R$5,000 = $2,279.14, R$7,000 = $3,190.79, R$10,000 = $4,558.28, R$15,000 = $6,837.41. As reported in the method section

d) refers to the proportional amount of savings each participant has in the end of a regular month (savings)

e) refers to the how much each participant fears that their savings would not last (duration); and

f) refers to the financial health index, which was calculated as (100-“Savings not last” + “Money savings”)/2. The financial health index describes the state of one's personal monetary affairs, hence, 0 depicts 0% of financial health and 100 depicts 100% of financial health. V = Cramer's V effect size; r = Pearson r effect size.

**Fig 4:**
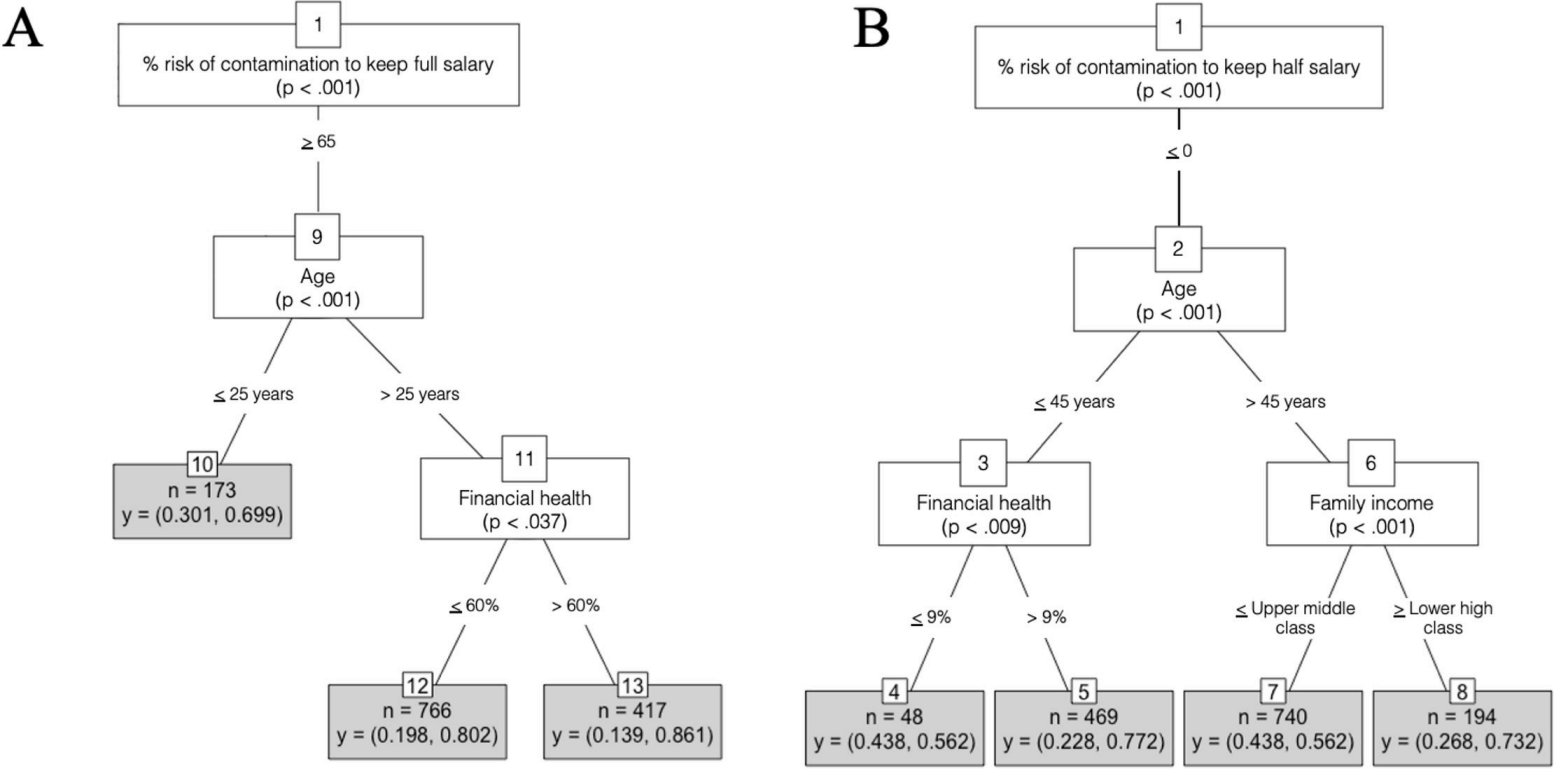
Conditional inference tree. A) RT group: participants who are more likely to be extreme risk-taking, i.e., who would always choose to leave home even for a half salary offer. B) RA group: participants who are more likely to be extreme risk-aversion, i.e., who would always choose to stay home even at the expense of a full salary offer.

The output from the conditional inference models was revealing. The first model (**Fig 4A**) aimed to identify those participants who are more likely to be part of the RT group. The model predicted that participants who tolerate a 65% chance of COVID-19 infection for a full salary offer (root node), are over 25 years old (node 11), and have a good financial health (over 60%), have an 86% chance of leaving home even for a half salary offer (terminal node 13). Nevertheless, this group represents 8% of the training sample (n = 417). Once the financial health is equal or lower than 60%, the model predicted that a similar participant would have an 6% less chance of leaving home even for a half salary offer (terminal node 12). The model then predicted that 173 participants (3% of the training sample) who tolerate a 65% chance of COVID-19 infection for a full salary offer and who are 25 years old or less, are the third group with a higher chance (69%) of leaving home even for a half salary offer (terminal node 10). In the test sample, the model proved to be able to distinguish participants who would always choose to leave home even for a half salary offer with a balanced accuracy of 94%, sensitivity of 93%, and specificity of 96% (Acc[.94] > NIR[.77], *p* < .000); Interrater reliability, Kappa = .84; *p* < .000).

The second model (**Fig 4B**) aimed to identify those participants who are more likely to be part of the RA group. The model predicted that participants who have a higher chance (77%) of staying home at the expense of any salary offer (terminal node 5) were those with who are not struggling concerning their financial health (node 3), are younger than 45 years old (node 2), and who did not tolerate even a minor chance of COVID-19 infection for a half salary offer (root node). This group represents 9% of the training sample (n = 469). Curiously, for a similar group of participants, but who have a poor financial health (lower than 9%, node 3), the chances of staying home at the expense of any salary offer decreases meaningfully for 56% (terminal node 4), although this group has only 48 participants. We also found that participants who are at the lower high class or higher (node 6), older than 45 years (node 2), and did not tolerate any chance of COVID-19 infection for a half salary offer (root node) have the second higher chance (73%) of staying home at the expense of any salary offer (terminal node 8). However, this group represents 3% of the training sample (n = 194). Once more, is worth mention that for a similar group of participants (14% of the training sample, n = 740), who are over than 45 years old, but from an upper middle class or lower, the chances of staying home at the expense of any salary offer decrease 17% (terminal node 7). In the test sample, the model proved to be able to distinguish participants who would choose to stay home even at the expense of a full salary offer with a balanced accuracy of 92%, sensitivity of 87%, and specificity of 97% (Acc[.89] > NIR[.80], *p* < .000); Interrater reliability, Kappa = .71; *p* < .000).

## Discussion

To our knowledge, our study represents a thus-far unique largescale experimental and multimodal investigation of decision-making behavior in the context of COVID-19, in which people from all over the world have been facing individual dilemmas with extensive social repercussions: to stay home with no risk of infection and possibly insufficient salary or to maintain an out-of-work routine, maintain their salary, and increase the possibility of infection. Here, we demonstrated that, in Brazil, the chance of COVID-19 infection that someone is willing to tolerate before abiding by social distancing recommendations at the expense of their salaries mainly depends on their risk perception, household income, and financial health. More precisely, we showed that people might intend to leave home to obtain their full salaries until the chance of COVID-19 infection reaches, on average, 38%, meaning that beyond this threshold people are more likely to stay home at the expense of their salary. Nevertheless, when the individual perceived risk of becoming ill from SARS-CoV-2 if infected is considered, this threshold drastically decreased to 13%, suggesting that people would prefer to stay home with no salary than face a 13% chance of falling severely ill. Remarkably, both the chance of COVID-19 infection that participants are willing to tolerate to leave home and guarantee their salaries, and the perceived risk of becoming ill from SARS-CoV-2 are mainly dependent on age, household income, and financial health. In this regard, we found that participants with a satisfactory financial health, older than 25 years and who tolerate a 65% chance of COVID-19 infection for a full salary offer are more likely to be part of the risk-taking group, who intend to leave home regardless of the COVID-19 infection level for obtaining even a half salary offer. Additionally, participants who are not struggling concerning their financial health, are younger than 45 years old, and who did not tolerate even a minor chance of COVID-19 infection for a half salary offer have a higher chance of being part of the risk-aversion group and, therefore, intending to remain home regardless of the salary offered.

Thus far, the inconsistency in implementing social distancing recommendations in Brazil has been based on the widespread assumption that many citizens who are not capable of adapting to home-office strategies may face severe income drawbacks and struggle with economic issues. Consequently, it has been supposed that those people with low socioeconomic status are more likely not to abide by social distancing recommendations, to avoid having insufficient resources such as food and basic needs [14, 15]. Nevertheless, by distinguishing household income from financial health, we were able to identify two opposite participant profiles that are not completely in accordance with the literature, which shows that the rate and burden of risk behaviors are affected by socioeconomic status, particularly for people living in low- and middle-income countries [31, 32].

At first, concerning the most likely risk-taking profile, we found that the higher the household income, the higher the risk of COVID-19 infection someone is willing to take to secure their monthly income, regardless of their educational level or even their financial health. This finding was surprising, because, at first glance, it is not in accordance with the literature mentioned above. However, one might hypothesize that these participants are not struggling with scarcity of resources—therefore, maintaining favorable financial health—precisely because they are willing to take risks and leave home to maintain their daily work routine. In this regard, a recent study that examine which socio-demographic characteristics predict self-protecting behaviors curiously found that higher income individuals were the most likely to permanently lose their jobs, despite also having the highest level of job security [33]. The authors, then, suggested that higher-income jobs are more secure in general, so a job loss reflects a large and permanent shift, e.g., a bankruptcy. If this statement proceeds, it might explain, at least in part, our findings concerning income and risk-taking behavior. Nevertheless, the same study discussed that lower-income respondents are more likely to lose their job or income due to the pandemic and, against our findings, self-protective behaviors, such social-distancing, are more and feasible for people with more income [33]. Another hypothesis is that other variables that we proved here to affect the tolerated risk of infection and risk perception, such as age and being part of the COVID-19 risk group, also contribute to the likelihood of having the risk-taking profile, even though this was not found in the CI models. This may be because the recursive partitioning algorithm applied in the CI models was unable to select the alternative input variables with strongest association to the target group and implement a binary split in the selected alternative input variable [28].

On the other hand, concerning the most likely risk-aversion profile, we found that the better the financial health, the lower the risk of COVID-19 infection someone is willing to take to secure their monthly income. This finding was expected, since evidence supports the fact that when facing strong emotions, such as those elicited by insufficient basic resources, people may place a relatively lower value on health consequences [1] and ignore the data regarding the probability and severity of a problem [3]. Specifically related with the temporal discounting paradigm, for instance, some evidence also suggests such an association. For example, frequent loan borrowers, who are predominantly people in the middle- and low-income brackets and with worse financial health, struggle to delay gratification [34]. In this sense, unfavorable socioeconomic status is usually associated with less delayed rewards [35–38], although some studies did not find such an association [39, 40]. In this sense, in general, people with low resources or who are struggling with scarcity are more likely to value small and immediate rewards, disregarding the long-term consequences of their choices [41].

Our study has several limitations that might be considered when interpreting the results. First, although we applied a series of criteria to maximize the reliability of the analyzed data—initially, by excluding participants with inconsistent demographic answers and, afterwards, by implementing an axiomatic approach to exclude those participants who were not maximizing their utility function—our data comprised an online survey and, therefore, it has its own methodological biases. For instance, we could not ensure that all participants provided accurate and honest answers, or that they felt comfortable providing answers that may present them in an unfavorable light, or even that they were alone and did not have environmental distractions when completing the survey. A second limitation concerns the specific period in which the data were collected, from April 18 to May 11. During these 24 days, the number of confirmed deaths in Brazil due to COVID-19 increased by 7204%, from 2,354 (mortality rate per 1,000 individuals = 0.011) to 169,594 (mortality rate per 1,000 individuals = 0.055) [42]. In this regard, our results still depict a specific moment of the outbreak, meaning that these outcomes, for example, may change during different phases of the pandemic period.

Furthermore, we highlight that our sample had a higher participation of women, although the strategy to promote the survey was targeting the general population (institutional websites announcements and calls and social network promotion targeting general population in Brazil). Despite that we noticed that others online surveys about COVID-19 also reported higher female participation [43]. Our study was conducted in Brazil, a country with continental dimensions that has been facing a political crisis that continues to increase with the rise of confirmed cases and deaths. In this regard, although our survey was spread throughout the country, most of our data were collected in southern Brazil, which differs from the northern region, both culturally and in terms of many sociodemographic factors. Due to a series of corruption scandals [44], the political leadership in Brazil has changed from a center-left orientation to a far-right orientation, which consequently reflects people’s beliefs and behavior. In this sense, it is plausible that participants who would always choose to leave home regardless of the chance of COVID-19 infection tend to support the current government, which has constantly diminished the risks and WHO recommendations, while those who would always choose to stay home regardless of salary offered tend to disagree with the current government. Indeed, a study has shown that after Brazil’s president emphatically dismissed the risks associated with the COVID-19 pandemic and advised against isolation, the social distancing measures taken by citizens in pro-government localities weakened compared to places where political support of the president is less strong [45].

Finally, although the 16 items of the Experimental COVID-19 Risk Aversion Questionnaire were randomized, the first level questions concerning household income, financial health and risk perception were not, and were always presented a priori. Therefore, it is possible that asking the perception of risk first and then the 16 items about COVID-19 risk aversion could have introduced some bias. Nevertheless, this possible bias was the same for all participants. Moreover, we did not inquire participants if they had a teleworkable occupation and whether they had basic infrastructure to do so (e.g., internet connection, room for homework, childcare necessities, etc.), even though this is an important factor accounting for the possibility of staying in home during the current pandemic scenario in large urban areas in Latin America [46]. Finally, as important as risk perception of getting severely ill, one may add that prosociality and generosity could also play an important role in leave/stay home decisions. For instance, it was shown that prosocial individuals are more likely to follow physical distancing guidelines, stay home when sick, and buy face masks [47]. Interestingly, the same study found that prosociality measured two years before the pandemic predicts health behaviors during the pandemic. Another study found a significant decrease on generosity in an experiment was conducted six days during the early pandemic [48]. The authors explained such reduction suggesting that the Covid-19 threat may decreased generosity toward the “outgroup” or toward people not considered as part of the “ingroup”. Nevertheless, more studies considering empathy and prosociality might be conducted in this regard.

Despite these limitations, our findings add to the political and public debate concerning lockdown strategies by demonstrating that, contrary to supposition, people with low socioeconomic status do not have a higher chance of ignoring social distancing recommendations due to personal economic matters, but precisely those people who have a comfortable socioeconomic status and who are not struggling with unfavorable financial health. Altogether, our data supports the necessity of social scientists to study behavior and the public policies to implement more effective measures, especially in such unexpected outbreaks. More precisely, it was shown that the burden of measures designed to stem the pandemic are unevenly distributed across sociodemographic groups and, therefore, it is necessary to shed light on what behavior can be expected of different segments of the population during a pandemic given heterogeneity in the incentives, constraints and circumstances people face [33]. Moreover, as showed before, high expected probability of running out of money is positively associated with mental health score, while perceived risks of dying from Covid-19 and social are also strongly correlated with higher levels of depression/anxiety [49]. Therefore, as the number of cases continues to rise, public-health politics may consider not only sensitizing people regarding infection rates, but also by providing health information and services, financial incentives, or fiscal alternatives so that people can maintain their businesses in a sustainable way.

## Supporting information

S1 File (DOCX)

S1 Data (CSV)

## References

[1] Leppin A, Aro AR. Risk perceptions related to SARS and avian influenza: theoretical foundations of current empirical research. Int J Behav Med. 2009;16(1):7–29. 10.1007/s12529-008-9002-8 19214752 PMC7090865

[2] Anderson LR, Mellor JM. Predicting health behaviors with an experimental measure of risk preference. J Health Econ. 2008;27(5):1260–74. 10.1016/j.jhealeco.2008.05.011 18621427

[3] Bavel JJV, Baicker K, Boggio PS, Capraro V, Cichocka A, Cikara M, et al Using social and behavioural science to support COVID-19 pandemic response. Nat Hum Behav. 2020 10.1038/s41562-020-0884-z 32355299

[4] Sohrabi C, Alsafi Z, O'Neill N, Khan M, Kerwan A, Al-Jabir A, et al World Health Organization declares global emergency: A review of the 2019 novel coronavirus (COVID-19). Int J Surg. 2020;76:71–6. 10.1016/j.ijsu.2020.02.034 32112977 PMC7105032

[5] Parmet WE, Sinha MS. Covid-19—The Law and Limits of Quarantine. N Engl J Med. 2020;382(15):e28 10.1056/NEJMp2004211 32187460

[6] Lau H, Khosrawipour V, Kocbach P, Mikolajczyk A, Schubert J, Bania J, et al The positive impact of lockdown in Wuhan on containing the COVID-19 outbreak in China. J Travel Med. 2020 10.1093/jtm/taaa037 32181488 PMC7184469

[7] Lal A, Moodie M, Peeters A, Carter R. Inclusion of equity in economic analyses of public health policies: systematic review and future directions. Aust N Z J Public Health. 2017 10.1111/1753-6405.12709 28898490

[8] Amuedo-Dorantes C, Borra C, Rivera-Garrido N, Sevilla A. Timing is Everything when Fighting a Pandemic: COVID-19 Mortality in Spain. IZA Institute of Labor Ecnomomics; 2020 10.1097/MLR.0000000000001300

[9] Standl F, Jöckel KH, Stang A. COVID-19 and the need of targeted inverse quarantine. Eur J Epidemiol. 2020 10.1007/s10654-020-00629-0 32328991 PMC7180640

[10] Croda J, Oliveira WK, Frutuoso RL, Mandetta LH, Baia-da-Silva DC, Brito-Sousa JD, et al COVID-19 in Brazil: advantages of a socialized unified health system and preparation to contain cases. Rev Soc Bras Med Trop. 2020;53:e20200167 10.1590/0037-8682-0167-2020 32320998 PMC7182282

[11] Ministry of Health B. Ministério da Saúde declara transmissão comunitária nacional. https://www.saude.gov.br/noticias/agencia-saude/46568-ministerio-da-saude-declara-transmissao-comunitaria-nacional; 2020.

[12] Ganem F, Mendes F, Oliveira S, Porto V, Araújo W, Nakaya H, et al The impact of early social distancing at COVID-19 Outbreak in the largest Metropolitan Area of Brazil.: medRxiv; 2020.

[13] Kirby J. Jair Bolsonaro undermined Brazil’s coronavirus response. Now there’s a political crisis. https://www.vox.com/2020/4/28/21228512/brazil-bolsonaro-coronavirus-moro: Vox; 2020.

[14] Datafolha. Opinião sobre a pandemia coronavírus. http://media.folha.uol.com.br/datafolha/2020/04/29/53099dbbcd7b05b8a943e4b6ed8a9802pand4.pdf; 2020.

[15] Meredith S. Could Bolsonaro be impeached? Brazil’s leader under intensifying pressure over coronavirus denial. https://www.cnbc.com/2020/04/03/coronavirus-brazils-bolsonaro-faces-calls-for-his-impeachment.html: CNBC; 2020.

[16] Kumar A, Priya B, Srivastava SK. Response to the COVID-19: Understanding implications of government lockdown policies. J Policy Model. 2020 10.1016/j.jpolmod.2020.09.001 33132465 PMC7588319

[17] Ferguson N, Laydon D, Nedjati-Gilani G, Imai N, Ainslie K, Baguelin M, et al Report 9: Impact of non-pharmaceutical interventions (NPIs) to reduce COVID-19 mortality and healthcare demand. 2020. 10.1007/s11538-020-00726-x PMC7140590 32270376

[18] Glimcher P, Feher E. Neuroeconomics: decision making and the brain 2 ed: Academic Press; 2013.

[19] CFPB. Measuring financial well-being: A guide to using the CFPB Financial Well-Being Scale 2015.

[20] CFPB. Getting started with measuring financial well-being: A toolkit for financial educators. 2019.

[21] CFPB. CFPB Financial Well-Being Scale: Scale develoment technical report. 2017.

[22] Brañas-Garza P, Jorrat D, Espín A, Sánchez A. Paid and hypothetical time preferences are the same: Lab, field and online evidence. Munich Personal RePEc Archive. 2020.

[23] Story GW, Vlaev I, Seymour B, Darzi A, Dolan RJ. Does temporal discounting explain unhealthy behavior? A systematic review and reinforcement learning perspective. Front Behav Neurosci. 2014;8:76 10.3389/fnbeh.2014.00076 24659960 PMC3950931

[24] Field A, Miles J, Field Z. Discovering Statistics Using R: SAGE Publication Ltd; 2012.

[25] Bollen KA, Noble MD. Structural equation models and the quantification of behavior. Proc Natl Acad Sci U S A. 2011;108 Suppl 3:15639–46 10.1073/pnas.1010661108 21730136 PMC3176611

[26] Kohn J, Bryant S. Factors leading to the U.S. housing bubble: a structural equation modeling approach. Research in Business and Economics Journal 2010.

[27] Thakkar J. Structural Equation Modeling: Application for research and practice (with AMOS and R). 1 ed: Springer Singapore; 2020.

[28] Hothorn T, Hornik K, Zeileis A. ctree: Conditional Inference Trees.

[29] Hothorn T, Zeileis A. partykit: A Modular Toolkit for Recursive Partytioning in R. Journal of Machine Learning Research. 2015;16:4.

[30] Kuhn M. Building Predictive Models in R Using the caret Package. Journal of Statistical Software. 2008;28(5):25.

[31] Abbasi-Ghahramanloo A, Heshmat R, Safiri S, Esmaeil Motlagh M, Ardalan G, Mahdavi-Gorabi A, et al Risk-Taking Behaviors in Iranian Children and Adolescents: A Latent Class Analysis Approach: Caspian IV Study. J Res Health Sci. 2018;18(4):e00428 30728314 PMC6941635

[32] Allen L, Williams J, Townsend N, Mikkelsen B, Roberts N, Foster C, et al Socioeconomic status and non-communicable disease behavioural risk factors in low-income and lower-middle-income countries: a systematic review. Lancet Glob Health. 2017;5(3):e277–e89. 10.1016/S2214-109X(17)30058-X 28193397 PMC5673683

[33] Papageorge N, Zahn M, Belot M, Broek-Altenburg E, Choi S, Jamison J, et al Socio-Demographic Factors Associated with Self-Protecting Behavior during the Covid-19 Pandemic.: National Bureau of Economic Research; 2020. 10.1007/s00148-020-00818-x PMC7807230 33462529

[34] Mahoney CT, Lawyer SR. Delay and probability discounting among payday and title loan recipients. Behav Processes. 2016;125:13–8. 10.1016/j.beproc.2016.01.011 26844664

[35] Ishii K. Subjective socioeconomic status and cigarette smoking interact to delay discounting. Springerplus. 2015;4:560 10.1186/s40064-015-1361-4 26435906 PMC4586184

[36] Reimers S, Maylor E, Stewart N, Chater N. Associations between a one-shot delay discounting measure and age, income, education and real-world impulsive behavior. Personality and Individual Differences. 2009;47(8).

[37] Harrison G, Lau M, Williams M. Estimating Individual Discount Rates in Denmark: A Field Experiment. American Economic Review. 2002;92(5).

[38] Stanger C, Ryan SR, Fu H, Landes RD, Jones BA, Bickel WK, et al Delay discounting predicts adolescent substance abuse treatment outcome. Exp Clin Psychopharmacol. 2012;20(3):205–12. 10.1037/a0026543 22182419 PMC3906638

[39] Pepper GV, Corby DH, Bamber R, Smith H, Wong N, Nettle D. The influence of mortality and socioeconomic status on risk and delayed rewards: a replication with British participants. PeerJ. 2017;5:e3580 10.7717/peerj.3580 28761784 PMC5530991

[40] Lindqvist A, Björklund F. How predictions of economic behavior are affected by the socio-economic status of the target person. J Soc Psychol. 2018;158(3):361–78. 10.1080/00224545.2017.1357527 28846063

[41] Griskevicius V, Tybur JM, Delton AW, Robertson TE. The influence of mortality and socioeconomic status on risk and delayed rewards: a life history theory approach. J Pers Soc Psychol. 2011;100(6):1015–26. 10.1037/a0022403 21299312 PMC3298774

[42] Guidotti E, Ardia D. COVID19. 2020.

[43] Jacques-Avino C, Lopez-Jimenez T, Medina-Perucha L, de Bont J, Goncalves AQ, Duarte-Salles T, et al Gender-based approach on the social impact and mental health in Spain during COVID-19 lockdown: a cross-sectional study. BMJ Open. 2020;10(11):e044617 10.1136/bmjopen-2020-044617 33234664 PMC7688440

[44] Economics. T. Brazil Corrpution Rank: Tranding Economics.; 2020 [Available from: https://tradingeconomics.com/brazil/corruption-rank.

[45] Ajzenman N, Cavalcanti T, Da Mata D. More than Words: Leaders’ Speech and Risky Behavior During a Pandemic. Pre print. 2020

[46] Berniell L, Fernandez D. Jobs’ amenability is not enough: The role of household inputs for safe work under social distancing in Latin American cities. Caracas: CAF 2020 10.1016/j.ehb.2020.100901

[47] Campos-Mercade P-, Meier A, Schneider F, Wengström E. Prosociality predicts health behaviors during the COVID-19 pandemic. University of Zurich: Department of Economics; 2020. 10.1016/j.jpubeco.2021.104367 PMC7842154 33531719

[48] Brañas-Garza P, Jorrat D, Alfonso-Costillo A, Espín A, Garcia T, Kovářík J. Exposure to the Covid-19 pandemic and generosity. MPRA Munich Personal RePEc Archive; 2020.

[49] Kampfen F, Kohler IV, Ciancio A, Bruine de Bruin W, Maurer J, Kohler HP. Predictors of mental health during the Covid-19 pandemic in the US: Role of economic concerns, health worries and social distancing. PLoS One. 2020;15(11):e0241895 10.1371/journal.pone.0241895 33175894 PMC7657497

