# Supplementary material for: A behavioral economic risk aversion experiment in the context of the COVID-19 pandemic
Source: PLoS One. 2021 Jan 19;16(1):e0245261. doi: 10.1371/journal.pone.0245261 (PMC7815159; doi:10.1371/journal.pone.0245261)
Supplement: S1 File — (DOCX) [file pone.0245261.s001.docx]

**A behavioral economic risk aversion experiment in the context of the COVID-19 pandemic**

**S1 File.**

| **Figure S1.** |
| --- |
|  |
| **Note.** GLM, Generalized Linear Model; SEM, Structural Equation Mode; CI, Conditional Inference Tree. |

| **Figure S2.** |
| --- |
|  |
| **Note.** Cumulative proportion was calculated based of the *k*’ estimate. For instance, a participant who chose to stay home when the risk of infection reached 35%, might have also chosen to stay home when the risk of infection was 5%, 10%, 15%, or 25%; but might not have chosen to stay home when the risk of infection was 50%, 65%, and 80%; so, we coded these behavior as 1, 1, 1, 1, 0, 0, 0, 0. Therefore, the cumulative proportion of staying home answers was obtained by the sum of staying home answers divided by the total amount of answers. |

| **Table S1.**  Demographics characteristics |  | |  |  |  |
| --- | --- | --- | --- | --- | --- |
|  | **Samples** | | **Test (df)** | **Effect size** | ***p*-value** |
|  | **Included**  **(n=7,216)** | **Non-included**  **(n=1,127)** |  |  |  |
| *Demographics* |  |  |  |  |  |
| Age, years range, n (%) |  |  | χ^2^=18.62 (4) | *V*=.03 | **.000** |
| 15-25 | 1211 (16.7) | 204 (18.1) | χ^2^=1.20 (1) | *V*=.01 | .272 |
| 26-35 | 2153 (28.8) | 310 (27.5) | χ^2^=2.54 (1) | *V*=.01 | .110 |
| 36-45 | 1874 (25.9) | 249 (22) | χ^2^=7.71 (1) | *V*=.03 | **.005** |
| 46-55 | 1060 (14.6) | 182 (16.1) | χ^2^=1.63 (1) | *V*=.01 | .200 |
| >56 | 918 (12.7) | 182 (16.1) | χ^2^=10 (1) | *V*=.03 | **.001** |
| Sex, n (%) |  |  | χ^2^=2.13 (1) | *V*=.01 | .144 |
| Female | 5412 (75) | 868 (77) | - | - | - |
| Male | 1804 (25) | 259 (22) | - | - | - |
| Unemployment status, n (%) | 266 (3.6) | 37 (3) | χ^2^=0.45 (1) | *V*=.00 | .501 |
| Health-related professional, n (%) | 2170 (30) | 381 (33.8) | χ^2^=6.40 (1) | *V*=.02 | **.011** |
| Education, n (%) | 778 (10.7) | 143 (12.6) | χ^2^=3.60 (1) | *V*=.02 | .057 |
| Secondary | 2360 (32.7) | 391 (34.6) | χ^2^=1.74 (1) | *V*=.02 | .186 |
| Undergraduate | 4078 (56.5) | 593 (52.6) | χ^2^=6.00 (1) | *V*=.01 | **.014** |
| Graduate | 632 (8.7) | 90 (7.9) | χ^2^=0.73 (1) | *V*=.02 | .391 |
| Flu-like Symptoms, n (%) | .37 (.66) | .38 (.66) | W=4058211 | *V*=.00 | .893 |
| Yes/no risk group for SARS-CoV-2, n (%) ^a^ | 4945 (68) | 754 (66) | χ^2^=1.18 (1) | *V*=.01 | **.**275 |
| Yes/no potential COVID-19 Contact, n (%) ^b^ | 5133 (71) | 800 (70) | χ^2^=0.01 (1) | *V*=.00 | .918 |
| *COVID-Risk Aversion Questionnaire* |  |  |  |  |  |
| Household income, n (%) ^c^ |  |  | χ^2^=20.11 (5) | *V*=.03 | **.001** |
| < R$3,000 (Lower low class) | 1155 (16) | 223 (19.7) | χ^2^=10.10 (1) | *V*=.03 | **.001** |
| R$3,001 - R$5,000 (Upper low class) | 1206 (16.7) | 219 (19.4) | χ^2^=5.08 (1) | *V*=.02 | **.024** |
| R$5,001 - R$7,000 (Lower middle class) | 1029 (14.2) | 156 (13.8) | χ^2^=0.13 (1) | *V*=.00 | .708 |
| R$7,001 - R$10,000 (Upper middle class) | 1124 (15.5) | 163 (14.4) | χ^2^=0.92 (1) | *V*=.01 | .335 |
| R$10,001 - R$15,000 (Lower high class) | 1070 (14.8) | 146 (12.9) | χ^2^=2.74 (1) | *V*=.01 | .097 |
| > R$15,001 (Upper high class) | 1632 (22.6) | 220 (19.5) | χ^2^=5.40 (1) | *V*=.02 | **.020** |
| Money savings, mean (SD) ^d^ | 44 (38) | 40 (36) | W=4268629 | r=.02 | **.006** |
| Savings not last, mean (SD) ^e^ | 45 (35) | 48 (35) | W=3794612 | r=.03 | **.000** |
| Financial health index, mean (SD) ^f^ | 50 (31) | 46 (30) | W=4357084 | r=.04 | **.000** |
| Perceived risk, mean (SD) | .38 (.26) | .40 (.25) | W=3894052 | r=.02 | **.021** |
| **Note.** a) Number of participants that fulfils, at least, one criterion for risk group for SARS-CoV-2. b) To present a more informative description of the sample, here we described the dichotomized version of the variable “Potential COVID-19 contact”. All participants who believe about being infected by COVID-19 or had/have past/current confirmed diagnosis of COVID-19 or believe about being close to someone infected by COVID-10 or had/have a relative’s past/current confirmed diagnosis of COVID-19 were classified as “yes, had potential COVID-19 contact”. Participants who reported no for all these questions were coded as “no, did not have potential COVID-19 contact”. c) For a matter of comparison with USA dollars using purchasing power parity function and based on The World Bank, household income can be estimated as following: R$3,000 = $1,367.48, R$5,000 = $2,279.14, R$7,000 = $3,190.79, R$10,000 = $4,558.28, R$15,000 = $6,837.41. As reported in the method section, d) refers to the proportional amount of savings each participant has in the end of a regular month (savings); e) refers to the how much each participant fears that their savings would not last (duration); and f) refers to the financial health index, which was calculated as (100-“Savings not last” + “Money savings”)/2. The financial health index describes the state of one's personal monetary affairs, hence, 0 depicts 0% of financial health and 100 depicts 100% of financial health. V = Cramer's V effect size; r = Pearson r effect size. | | | | | |

**Experimental COVID-19 Risk-Aversion Questionnaire**

To certify that people behaved as expected on the experimental COVID-Risk Aversion Questionnaire, independently of any additional sociodemographic variable, a series of generalized linear mixed models (GLMM) were performed. Thus, the GLMM included all the questions that comprises the first and the second level of the COVID-Risk Aversion Questionnaire (i.e., risk perception of becoming ill from the SARS-CoV-2, household income, salary offered and risk of COVID-19 contamination) as fixed predictors and the dichotomic variable stay or leave home as the outcome. The GLMM was performed in a long format version of the dataset, in which each participant is represented 16 lines and each line represents one item of the COVID-Risk Aversion Questionnaire. The GLMM requires a random effect for each individual, because the data is nested among participants and observations are not independent of one another(1). The reason to use this analytical strategy is because it comes close to experimental neuroeconomic approaches(2, 3) in which the decision behavior is observed item-by-item in its simplest form.

As shown in **Table S2**, five models were tested. At first, to explore what degree of risk of COVID-19 infection is necessary so that people intend to stay home, even when doing so means losing their salary, a GLMM including only the manipulated chance of COVID-19 infection as fixed predictor, and the dichotomic variable stay (1) or leave (0) home as the outcome was performed (model 1). Next, a similar model was performed adding the offered salary as fixed predictor (model 2). Then, because both chance of COVID-19 infection and the salary offered were manipulated among the 16 items of the COVID-19 Risk Aversion Questionnaire, an interaction between these variables was considered (model 3). The model with the interaction proved to better fit the data when compared to the previous one without the interaction (*x*^2^[1]=64.28, *p*<.0001) (**Table S2**). Model 3 revealed that both the manipulated chance of COVID-19 infection (β =-15.83, z=-57.94, *p*<.000), the salary offered (β =2.27, z=41.04, *p*<.000), and their interaction (β =-0.77, z=-5.78, *p*<.000) influence participants’ decision-making. This result suggests, as expected, that as the chance of COVID-19 infection increases, the likelihood of a participant leaving home decreases, regardless of the salary offered. Nevertheless, when the full salary was offered instead of half salary, the likelihood of a participant leaving home increased. The model also showed that fixed effects explained 19% of the variance (marginal R^2^), while 96% of the variance was explained by both the fixed and random effects (conditional R^2^).

Next, to investigate how individual risk perception of becoming severely ill if infected by COVID-19 influences leaving home intention, we performed a fourth GLMM just like the previous one but including risk perception as an additional fixed predictor (model 4). The model revealed a significant effect for risk perception (β =-8.43, z=-18.54, *p*<.000), and the effects for the chance of COVID-19 infection (β =-15.85, z=-59.06, *p*<.000), the salary offered (β =2.27, z=41.20, *p*<.000), and their interaction (β =-0.77, z=-5.87, *p*<.000) remained significant. This finding indicates that as the perceived risk of becoming severely ill increases, the likelihood of a participant leaving home to guarantee monthly income decreases, as expected. Finally, because household income is also part of the COVID-19 Risk-Aversion Questionnaire, it was included in model 5. The results were similar as the previous one, but in addition, an effect for household income was also found (β =.68, z=10.88, *p*<.000), suggesting that, in general, as the income increases there the likelihood of a participant leaving home to guarantee monthly income increase as well. Furthermore, the model with all items of the COVID-19 Risk-Aversion Questionnaire proved significantly better than the previous ones, increasing the marginal R^2^ to .25 (conditional R^2^=.96), suggesting that 25% of the variance was mainly explained by the predictors. Altogether, these findings provided an important insight about how people might behave in the experimental COVID-Risk Aversion Questionnaire, independently of any sociodemographic variable.

| **Table S2.**  Experimental COVID-19 Risk-Aversion Questionnaire: generalized linear mixed models. | | | | | | | | |
| --- | --- | --- | --- | --- | --- | --- | --- | --- |
|  | **Contamination risk** | **Salary offered** | **Interaction ^a^** | **Risk perception** | **Household income** | **Marginal R^2^** | **Model comparison** | ***p*-value** |
| Null model | - | - | - | - | - | - | - | - |
| Model 1 | -14.47 *** | - | - | - | - | .187 | 1 vs. 0 | **<.0001** |
| Model 2 | -17.05 *** | 2.02 *** | - | - | - | .198 | 2 vs. 1 | **<.0001** |
| Model 3 | -15.83 *** | 2.27 *** | -0.77 *** | - | - | .199 | 3 vs. 2 | **<.0001** |
| Model 4 | -15.85 *** | 2.27 *** | -.77 *** | -8.43 *** | - | .241 | 4 vs. 3 | **<.0001** |
| Model 5 | -15.85 *** | 2.27 *** | -.78 *** | -7.77 *** | .68 *** | .255 | 5 vs. 3 | **<.0001** |

| **Table S3.**  Structural Equation Modeling: Main outcome. | | | | |
| --- | --- | --- | --- | --- |
|  | Estimate | SE | z-value | p-value |
| **Regressions** |  |  |  |  |
| *k at half salary* |  |  |  |  |
| Risk perception | -0.19 | 0.016 | -12.27 | <0.001 |
| Household income | 0.03 | 0.002 | 13.57 | <0.001 |
| Financial Health | -0.11 | 0.013 | -8.96 | <0.001 |
| Education | 0.00 | 0.006 | 0.77 | 0.440 |
| Age | -0.01 | 0.003 | -4.09 | <0.001 |
| Sex | -0.00 | 0.008 | -0.90 | 0.366 |
| Risk group | -0.01 | 0.006 | -2.47 | 0.013 |
| Flu-like symptoms | -0.03 | 0.013 | -2.36 | 0.018 |
| COVID-19 contact | 0.03 | 0.006 | 7.07 | <0.001 |
| Health professional | 0.00 | 0.008 | 0.83 | 0.406 |
| *k at full salary* |  |  |  |  |
| Risk perception | -0.20 | 0.016 | -13.12 | <0.001 |
| Household income | 0.02 | 0.002 | 10.57 | <0.001 |
| Financial Health | -0.12 | 0.012 | -10.37 | <0.001 |
| Education | 0.01 | 0.006 | 2.09 | 0.036 |
| Age | -0.02 | 0.003 | -6.81 | <0.001 |
| Sex | -0.01 | 0.008 | -2.19 | 0.028 |
| Risk group | -0.01 | 0.006 | -2.49 | 0.013 |
| Flu-like symptoms | -0.02 | 0.013 | -2.21 | 0.027 |
| COVID-19 contact | 0.03 | 0.005 | 6.63 | <0.001 |
| Health professional | 0.01 | 0.008 | 1.32 | 0.185 |
| **Covariances** |  |  |  |  |
| *Risk* *perception* |  |  |  |  |
| Financial Health | -0.01 | 0.001 | -13.67 | <0.001 |
| Household income | -0.07 | 0.005 | -15.00 | <0.001 |
| Sex | 0.01 | 0.001 | 8.78 | <0.001 |
| Risk group | 0.06 | 0.002 | 34.27 | <0.001 |
| *Household* *income* |  |  |  |  |
| Financial Health | 0.18 | 0.007 | 28.72 | <0.001 |
| Age | 0.51 | 0.024 | 21.44 | <0.001 |
| Education | 0.35 | 0.015 | 24.55 | <0.001 |
| Sex | -0.07 | 0.008 | -9.82 | <0.001 |
| *Financial* *Health* |  |  |  |  |
| Education | 0.01 | 0.002 | 6.71 | <0.001 |
| *Education* |  |  |  |  |
| Age | 0.22 | 0.01 | 22.66 | <0.001 |
| *Sex* |  |  |  |  |
| Health professional | 0.02 | 0.002 | 11.49 | <0.001 |
| *Age* |  |  |  |  |
| Risk group | 0.23 | 0.009 | 27.23 | <0.001 |
| *COVID-19 contact* |  |  |  |  |
| Health professional | 0.06 | 0.004 | 16.77 | <0.001 |
| *k at half salary* |  |  |  |  |
| k at full salary | 0.08 | 0.001 | 57.27 | <0.001 |

**Reference**

1. Finch W, Bolin J, Kelley K. Multilevel Modeling Using R. 2nd ed: Chapman and Hall/CRC; 2019.

2. Caplin A, Glimcher P. Basic Methods from Neoclassical Economics. In: Glimcher P, Fehr E, editors. Neuroeconomics, Decision Making and the Brain. 2 ed: Elsevier; 2014.

3. Glimcher P, Feher E. Neuroeconomics: decision making and the brain. 2 ed: Academic Press; 2013.
